# A rare cause of a right atrial mass

**DOI:** 10.1186/1749-8090-10-S1-A80

**Published:** 2015-12-16

**Authors:** Ho-fon Royce Law, Rory Beattie, Alastair Graham

**Affiliations:** 1 Queen's University Belfast, University Road, Belfast, BT7 1NN, UK; 2 Cardiothoracic Surgery Department, Royal Victoria Hospital, Belfast, BT12 6BA, UK

## Background/Introduction

This 62 year-old gentleman presented with increasing shortness of breath on exertion and reduced exercise tolerance (NYHA class III). Brain natriuretic peptide and D-dimer were markedly elevated. ECG showed no evidence of acute ischaemia. There was a history of gastro-oesophageal reflux disease and excessive alcohol consumption 8 years previously. He was an ex-smoker with a 20 pack year history.

## Aims/Objectives

CT-PA was performed to investigate for possible pulmonary embolism. A 78 × 51 mm right atrial mass was identified with associated pericardial and pleural effusions. Transthoracic Echo confirmed mass was prolapsing across tricuspid valve into right ventricle. CT also showed liver cirrhosis with a large mass within segments IVa and VIII which extended into the middle hepatic vein, inferior vena cava and right atrium.

## Method

After median sternotomy cardiopulmonary bypass was instituted with arterial cannula via the ascending aorta and venous cannulae via superior vena cava and right femoral vein. Cannulation of the inferior vena cava was avoided as it was obstructed by the mass. Superior vena cava and inferior vena cava were then snugged.

The procedure was performed without cardioplegia arrest (on-pump beating heart): right atrium opened; tumour inspected and delivered. The right ventricle was flushed out with saline whilst the main pulmonary artery was compressed.

## Results

The delivered tumour weighed 93g and measured 86 × 62 × 39 mm. Morphological features of which are consistent with metastatic hepatocellular carcinoma. There was a significant fall in central venous pressure post-operation, from 29 mmHg to 11 mmHg. Patient was admitted to HDU and extubated in 3 hours. He was discharged home two weeks post-operatively after having a 20kg diuresis. His hepatocellular carcinoma is managed palliatively with prognosis between 3 and 6 months. He is currently being considered for biological treatment with sorafenib.

## Discussion/Conclusion

Although a rare cause of an intra-cardiac mass, metastatic hepatocellular tumours can be treated similarly to renal cell carcinoma tumours of the heart. Surgical removal of the mass is an effective palliative procedure to improve quality of life. Communication with patients can be difficult as these heart tumours can be the initial presentation of metastatic tumour.

## Consent

Written informed consent was obtained from the patient for publication of this abstract and any accompanying images. A copy of the written consent is available for review by the Editor of this journal

